# Cyclosporine A Modulates LSP1 Protein Levels in Human B Cells to Attenuate B Cell Migration at Low O_2_ Levels

**DOI:** 10.3390/life12081284

**Published:** 2022-08-22

**Authors:** Shannon P. Hilchey, Mukta G. Palshikar, Eric S. Mendelson, Shichen Shen, Sailee Rasam, Jason A. Emo, Jun Qu, Juilee Thakar, Martin S. Zand

**Affiliations:** 1 Department of Medicine, Division of Nephrology, University of Rochester Medical Center, Rochester, NY 14642, USA; 2 Biophysics, Structural, and Computational Biology Program, University of Rochester Medical Center, Rochester, NY 14642, USA; 3 Department of Pharmaceutical Sciences, State University of New York (SUNY) at Buffalo, Buffalo, NY 14203, USA; 4 New York State Center of Excellence in Bioinformatics & Life Sciences, State University of New York (SUNY) at Buffalo, Buffalo, NY 14203, USA; 5 Department of Microbiology and Immunology, University of Rochester Medical Center, Rochester, NY 14642, USA; 6 Department of Biostatistics and Computational Biology, University of Rochester Medical Center, Rochester, NY 14642, USA; 7 Clinical and Translational Science Institute, University of Rochester Medical Center, Rochester, NY 14642, USA

**Keywords:** human B cells, chemotaxis, CXCR4, LSP1

## Abstract

Coordinated migration of B cells within and between secondary lymphoid tissues is required for robust antibody responses to infection or vaccination. Secondary lymphoid tissues normally expose B cells to a low O${}_{2}$ (hypoxic) environment. Recently, we have shown that human B cell migration is modulated by an O${}_{2}$-dependent molecular switch, centrally controlled by the hypoxia-induced (transcription) factor-1α (HIF1A), which can be disrupted by the immunosuppressive calcineurin inhibitor, cyclosporine A (CyA). However, the mechanisms by which low O${}_{2}$ environments attenuate B cell migration remain poorly defined. Proteomics analysis has linked CXCR4 chemokine receptor signaling to cytoskeletal rearrangement. We now hypothesize that the pathways linking the O${}_{2}$ sensing molecular switch to chemokine receptor signaling and cytoskeletal rearrangement would likely contain phosphorylation events, which are typically missed in traditional transcriptomic and/or proteomic analyses. Hence, we have performed a comprehensive phosphoproteomics analysis of human B cells treated with CyA after engagement of the chemokine receptor CXCR4 with CXCL12. Statistical analysis of the separate and synergistic effects of CyA and CXCL12 revealed 116 proteins whose abundance is driven by a synergistic interaction between CyA and CXCL12. Further, we used our previously described algorithm BONITA to reveal a critical role for Lymphocyte Specific Protein 1 (LSP1) in cytoskeletal rearrangement. LSP1 is known to modulate neutrophil migration. Validating these modeling results, we show experimentally that LSP1 levels in B cells increase with low O${}_{2}$ exposure, and CyA treatment results in decreased LSP1 protein levels. This correlates with the increased chemotactic activity observed after CyA treatment. Lastly, we directly link LSP1 levels to chemotactic capacity, as shRNA knock-down of LSP1 results in significantly increased B cell chemotaxis at low O${}_{2}$ levels. These results directly link CyA to LSP1-dependent cytoskeletal regulation, demonstrating a previously unrecognized mechanism by which CyA modulates human B cell migration. Data are available via ProteomeXchange with identifier PXD036167.

## 1. Introduction

The elicitation of B cell immune responses to either infection or vaccination occurs predominately within secondary lymphoid tissues, such as the draining lymph nodes (LN). Physiological O${}_{2}$ levels within LN range from 1% to 5% [1,2], significantly lower than traditional tissue culture conditions (≈19% for a humidified and 5% CO${}_{2}$ incubator) frequently used to study B cell responses in vitro, an often overlooked aspect of B cell immunology. Consequently, hypoxia, a low O${}_{2}$ environment, is increasingly recognized as a key immune modulator, mediated in lymphocytes by the transcription factor HIF-1α. We have shown that HIF-1α transcripts are upregulated after unadjuvanted influenza vaccination in differentiating B cells in vitro and migrating human plasma cells after vaccination in vivo [3]. In addition, our work has shown that cyclosporine A (CyA) directly alters B cell chemokine receptor responsiveness (CXCR4/CXCR5) via HIF-1α, a previously unknown mechanism of CyA action, directly modulating B cell function [3]. These findings suggest that the physiological O${}_{2}$ levels encountered in vivo are critical for B cell function, and that perturbations in either O${}_{2}$ levels themselves, or modulation of HIF-1α signaling, may profoundly alter B cell immune responses.

Coordinated B cell migration is dependent on chemokine receptor signaling in response to local chemokine gradients. Signaling by the CXCR4 and CXCR5 chemokine receptors in response to ligand gradients (CXCL12 and CXCL13, respectively) is critical for B cell migration between and within secondary lymphoid organs, especially within developing germinal centers (GC). Current data indicates that the O${}_{2}$ responsive transcription factor HIF-1α is a central regulator of this process, and is regulated by local O${}_{2}$ levels [4,5,6]. B cell location with respect to CD4 T and antigen-presenting cells within the GC is highly dependent on CXCR4 and CXCR5 modulation [7]. Importantly, perturbation of chemokine signaling disrupts the spatial localization of B cells between GC light and dark zones, decreasing somatic hypermutation and Ig affinity maturation [7]. In addition, low O${}_{2}$ tension is required for the generation of high-affinity, somatically hypermutated antibodies [4]. Consistent with these observations, proximity to nodal blood vessels and their higher O${}_{2}$ levels impairs development of B cell follicle into GC [6]. This evidence shows that GC O${}_{2}$ tension and coordinated cellular migration are tightly regulated and critical aspects of B cell responses.

Calcineurin inhibitors (CNIs) such as cyclosporine (CyA) are used to reduce allograft rejection in solid-organ transplant recipients [8]. A side effect of CNI therapy is reduced B cell responsiveness and low IgG levels, including after vaccination against influenza and other pathogens [9,10,11]. CNIs inhibit calcineurin signaling in T cells by preventing calcineurin-dependent dephosphorylation of the transcription factor NFAT, inhibiting IL-2 production and T cell proliferation [12]. Thus, CNIs are thought to primarily suppress CD4 T cell help, indirectly causing B cell hyporesponsiveness [12]. In contrast, we have shown that CNIs directly alter B cell CXCR4/CXCR5 responsiveness via HIF-1α, a previously unknown mechanism for modulating B cell function [3]. These results provide strong support for the hypothesis that CNIs modulate HIF-1α responses directly in B cells, altering chemokine dependent migration of B cells, impairing GC responses, and resulting in decreased antibody production in vivo. The mechanisms by which O${}_{2}$ levels alter B cell chemotactic responses, and how CNIs modify this response, remain largely undefined. However, proteomics analysis suggests that cytoskeletal components have a mechanistic role in this pathway [3].

Cytoskeletal rearrangement is required for cellular migration [13]. Several cytoskeletal proteins link the B cell cortical actin network to the plasma membrane, including myosin. Structural changes in cortical actin mediate BCR activation and chemotaxis in response to chemokine gradients [14]. The actin cytoskeleton contains more than a hundred different actin binding proteins, including important structural regulators such as LSP1 (lymphocyte specific protein 1) [15,16]. LSP-1 binding to f-actin plays a direct role in cortical actin filament polymerization and dynamic structural changes affecting cellular migration [17], making it a potential molecular mediator of hypoxia-induced changes in B cell motility. Indeed, LSP1 was initially identified as a marker of neutrophil migratory dysfunction. LSP-1 overexpression in neutrophils results in impaired chemotactaxis, directly linking LSP1 to cellular migratory capacity [16,18,19,20]. In addition, hypoxia also alters cytoskeletal rearrangement [21]. These obersvations suggest that O${}_{2}$ levels affect B cell migratory capacity through calcineurin-pathway-modulated cytoskeletal rearrangement via transcriptional, post-translational means as well as phosphorylation events.

In this report, we use discrete state modeling [22] of phosphoproteomics data to specifically identify CyA-modulated cytoskeletal components that play a direct role in attenuating B cell migration at low O${}_{2}$ levels. Indeed, our analysis identified LSP1 as the cytoskeletal protein that was the downstream modulator of CXCL12/CXCR4-mediated B cell migration, linking the HIF-1α, CXCR4 and CyA-sensitive calcineurin signaling pathways. These findings were confirmed with experimental data showing an inverse correlation between LSP1 protein levels and O${}_{2}$ levels, with CyA treatment resulting in decreased LSP1 levels at low O${}_{2}$. In addition, a targeted shRNA knockdown of LSP1 resulted in a significant increase in B cell chemotactic activity at low O${}_{2}$ levels. Taken together, these data suggest LSP1 as an integrator of HIF-1α, CXCR4 and calcineurin signaling pathways.

## 2. Materials and Methods

### 2.1. Human Cell Lines

Human cell line: RAMOS (ATCC, Manassas, VA, USA; CRL-1596) Burkitt’s lymphoma B cell line was maintained in complete RPMI 1640 media supplemented with 10% FBS (cR10; Invitrogen, Carlsbad, CA, USA).

### 2.2. O${}_{2}$ Controlled Experiments

RAMOS B cells were resuspended in cR10 media at $5\times 10^{4}$ cell/mL and added to appropriate-sized tissue culture plates or flasks depending on total volume. To select flasks, CyA (Sigma-Aldrich, St. Louis, MO, USA) was added at the concentration indicated in the text and/or figure legends. Plates or flasks were then placed within controlled environment C-chamber 37 °C, 5% CO${}_{2}$ incubator inserts (Biospherix, Parish, NY, USA) that were incubated for 24 h at the indicated O${}_{2}$ levels or placed within a traditional 5% CO${}_{2}$, 37 incubator (19% O${}_{2}$). After 24 h, cells were removed and analyzed by either chemotaxis, western blot or phosphoproteomics assays as described below.

### 2.3. Chemotaxis Assay

Chemotaxis assays were performed as previously described [3]. Briefly, RAMOS human B cells were resuspended in freshly prepared migration media (RPMI + 1% BSA + 100 U/mL Pen/Strep), incubated for 1 h, and $5\times 10^{5}$ cells (in 75 µL ) loaded into the upper chambers of 5 µm polycarbonate 96-well transwell plates (Corning, Corning, NY, USA). In triplicate wells, migration media containing varying concentrations of CXCL12 (0 ng/mL to 500 ng/mL) were added to the bottom chambers of the transwells (235 µL per well). Plates were incubated for 1 h at varying O${}_{2}$ levels as described above. Upper transwells were removed and 100 µL of Cell Titer Glo (Promega, Madison, WI, USA) added to each bottom well; plates were then placed on an orbital shaker for 2 min and incubated at RT for 10 min in the dark. Luminescence was measured on a SynergyTM HT microplate reader (BioTek Instruments, Winooski, VT, USA) and relative luminescent units (RLU) reported.

### 2.4. HIF-1α and LSP1 Western Blots

HIF-1α Western blots were performed on nuclear lysates prepared from cell pellets using NE-PER nuclear and cytoplasmic extraction kits (Thermo Fisher Scientific, Waltham, MA, USA) as previously described [3]. Western blotting for LSP1 was performed using whole-cell lysates prepared with RIPA lysis and extraction buffer, as recommended by the manufacturer (Thermo Fisher Scientific, Waltham, MA, USA). Proteins were resolved on precast NuPAGE 4–12% Bis-Tris protein gels (Invitrogen, Carlsbad, CA, USA) and transferred to a PVDF membrane (Bio-Rad, Hercules, CA, USA). The membrane was then blocked with 1X TBS-T (Tris-Buffered Saline (Bio-Rad, Hercules, CA, USA)) with 0.05% Tween-20 (Sigma-Aldrich, St. Louis, MO, USA) + 5% blotting grade nonfat dry milk (Bio-Rad, Hercules, CA, USA) for 1 h at room temperature on a rocker or overnight at 4 °C. Blots were then probed with a mouse antihuman LSP1 primary antibody (Santa Cruz Biotechnology, Dallas, TX, USA) at a dilution of 1:200 in TBS-T + 5% milk or a mouse antihuman HIF-1α primary antibody at a 1:250 dilution in TBS-T + 5% milk. As a loading control, blots were also probed with a 1:5000 dilution mouse antiactin primary antibody (BD Biosciences, San Diego, CA, USA). For both LSP1 and actin detection, a horse radish peroxidase (HRP) conjugated goat antimouse IgG secondary antibody (BD Biosciences, San Diego, CA, USA) was used at a dilution of 1:1000 in TBS-T + 5% milk, and blots were incubated for 1 h at room temp on a rocker. Blots were washed 3X with TBS-T and incubated on a rocker at room temperature for 5 min with freshly prepared Enhanced Chemiluminescence (ECL) substrate (Thermo Fisher Scientific, Waltham, MA, USA). Blots were imaged on a ChemiDoc MP imaging system. Densitometry was performed using Image Lab software version 6.0.1 (Bio-Rad, Hercules, CA, USA).

### 2.5. Statistical Analysis of Chemotaxis and Western Experiments

Chemotaxis data from 4 independent experiments were analyzed using two-way Anova and *p* values corrected for multiple comparisons using the Bonferroni adjustment (Prism v8.1.0, San Diego, CA, USA). For the Western blot data, band densities from 3 independent experiments were analyzed using paired *t*-tests (Prism v8.1.0, San Diego, CA, USA).

### 2.6. shRNA Lentiviral Transfection

RAMOS cells were transfected using LSP1 shRNA or control shRNA lentiviral particles according to the manufacturer’s recommendations (Santa Cruz Biotechnology, Dallas, TX, USA). RAMOS cells at $2.5\times 10^{5}$ cells/mL in cR10 media were seeded in 12-well cell culture plates and incubated for 24 h at 37 and 5% CO${}_{2}$. The following day, the cells were pelleted at 250 g and the cell pellets resuspended in 1 mL of cR10 media containing 5 µg/mL of polybrene. The resuspended cells were added back to their original wells with 5 µg/mL of either control or LSP1 shRNA lentviral particles (along with a no-LV particles control). Plates were gently swirled and placed back in the incubator for 24 h. On the third day, cells were pelleted to remove the polybrene-containing media, resuspended in 1 mL of fresh cR10 media, added to new 12-well cell culture plates, and incubated for an additional 24 h. The contents of each well were transferred to a T-25 cell culture flask with 4 mL of cR10 media and incubated for an additional 46 h. After the incubation period, the contents of each T-25 flask were pelleted and resuspended in 15 mL of cR10 media containing 0.5 µg/mL of puromycin in T-75 flasks to select for puromycin-resistant cells. Cells were maintained and expanded in puromycin-containing media until all of the cells in the control no-LV patricles flasks had completely died. Multiple aliquotes from both the LSP1 and control shRNA transductions were frozen in 90% FBS + 10% DMSO. Aliquotes were stored in a liquid nitrogen cryo-freezer and thawed as needed for experiments. Thawed cells were maintained in puromycin containing cR10 media.

### 2.7. Phosphoproteomics

#### 2.7.1. RAMOS Cell Sample Preparation

RAMOS cells were resuspended in cR10 media at $5\times 10^{5}$ cells/mL to a final volume of 10 mL in T25 tissue culture flasks (BD Biosciences, San Diego, CA USA) and either left untreated or treated with cyclosporine A (Sigma-Aldrich, St. Louis, MO USA) at 1 µg/mL. Pairs of untreated or CyA treated cells were then incubated in either 19% or 1% O${}_{2}$ chambers for 24 h. Cells were then rapidly harvested into 50 mL conical tubes (BD Biosciences, San Diego, CA USA) containing 30 mL of ice cold PBS. Cells were pelleted and washed three times in 10 mL ice cold PBS. After the final wash, cells were resuspended in 1 mL ice cold PBS and transferred to 1.5 mL microcentrifuge tubes and pelleted. Supernatants were removed and cell pellets flash frozen in liquid nitrogen and stored at −80 °C until lysed for analysis.

#### 2.7.2. Protein Sample Preparation

Cell lysis and protein digestion procedures were adopted and slightly modified from a previously published surfactant cocktail-aided extraction/precipitation/on-pellet digestion protocol [23]. Pelleted cells were lysed in 8M urea, 50mM HEPES buffer at pH 8.5 supplemented with complete protease and PhosSTOPphosphatase inhibitor cocktails tablets (Roche Applied Science, Indianapolis, IN, USA). Samples were vortexed and placed on ice for 30 min, and then sonicated using a high-energy probe (3–4 sonication-cooling cycles, 20 s each). Sonicated samples were then centrifuged at 20,000× *g* under 4 ${}^{\circ }$C for 30 min, and supernatant was transferred to new Eppendorf tubes. Sample protein concentrations were determined by bicinchoninic acid (BCA) assay, and 2 mg protein lysates were aliquoted for protein digestion. Protein lysates were sequentially reduced with 10 mM dithiothreitol (DTT) for 1 h and alkylated by 25 mM iodoacetamide (IAM) for 30 min. Both steps were performed under agitation at 37 ${}^{\circ }$C in a covered thermomixer (Eppendorf, Enfield, CT, USA). Protein was then pelleted by three-phase precipitation (methanol:chloroform:water:sample = 4:1:3:1) and centrifugation (20,000× *g*, 4 ${}^{\circ }$C, 30 min), and the pellets were gently washed in 10 mL methanol. After centrifugation (20,000× *g*, 4 ${}^{\circ }$C, 30 min), pelleted protein was air-dried for 1 min and was resuspended in 2 mL 50 mM HEPES pH 8.5 (Santa Cruz Biotechnology, Inc., Dallas, TX, USA). Proteomics-grade Trypsin (MilliporeSigma, Burlington, MA, USA) at a concentration of 0.25 mg/mL (dissolved in 50 mM HEPES, pH 8.5) was added to each sample to reach a final enzyme:substrate ratio of 1:20, and samples were incubated under 37 ${}^{\circ }$C overnight (∼16 h) with constant shaking for proteolytic digestion. Samples were then acidified by 0.5% trifluoroacetic acid (TFA) to inactivate trypsin, and derived peptides were subjected to desalting using Oasis PRiME HLB Vac Cartridges (Waters, Milford, MA, USA).

#### 2.7.3. Phosphopeptide Enrichment

Enrichment of phosphopeptides was accomplished based on a titanium dioxide (TiO${}_{2}$) bead-based protocol established by Paulo JA et al. with minor modifications [24]. Desalted tryptic peptides (derived from 2 mg protein for each sample) were first reconstituted in 500 µL 2M lactic acid/50% acetonitrile (ACN)/1% TFA and centrifuged at 20,000× *g* under 4 ${}^{\circ }$C for 20 min. Supernatant was transferred to Eppendorf tubes containing 12 mg 5 µm Titanosphere TiO${}_{2}$ beads (GL Biosciences, Tokyo, Japan), and then incubated for 2 h (1 h vortexing, 1 h end-over-end rotation) at room temperature. TiO2 beads were then sequentially washed with: (1) 1 mL 2M lactic acid/50% ACN, 3X; (2) 1 mL 50% ACN/0.1% TFA, 3X; and (3) 1 mL 25% ACN/0.1% TFA, 3X. Phosphopeptides were then eluted with 200 µL 300 mM ammonium hydroxide (NH${}_{4}$OH) once and 300 µL 700 mM NH${}_{4}$OH twice. All elution steps were performed on 0.45-micron spin filters (MilliporeSigma, Burlington, MA, USA) at 100× *g*. Eluted peptides were acidified by FA to a final concentration of 5%, desalted using Oasis PRiME HLB Vac Cartridges, dried using a SpeedVac concentrator (Thermo Fisher Scientific, Rockford, IL, USA), and reconstituted in 100 µL 50 mM HEPES (pH 8.5).

#### 2.7.4. Phosphopeptide Labeling

Ten-plex Tandem Mass Tag (TMT) reagents (Thermo Fisher Scientific, Rockford, IL, USA) were used to achieve isobaric labeling of the enriched phosphopeptides. Briefly, 0.8 mg TMT reagents were dissolved in 41 µL anhydrous ACN, and 4 µL was added to each phosphopeptide samples. Samples were incubated at room temperature for 1 h with constant shaking. The reaction was terminated by the addition of 3.2 µL 5% hydroxylamine and incubated for 15 min. Ninety-five µL labeled phosphopeptide from each sample/TMT channel was combined for high-pH reversed-phase fractionation. Another 2 µL from each sample/TMT channel was combined to check TMT labeling efficiency.

#### 2.7.5. Peptide Fractionation

TMT-labeled phosphopeptides were first dried by a SpeedVac concentrator, reconstituted in 300 µL 0.1% TFA, and loaded onto conditioned reversed-phase fractionation spin columns (Thermo Fisher Scientific, Rockford, IL, USA). The column was washed with 300 µL 5% ACN/0.1% triethylamine (TEA), and the peptides were sequentially eluted by 10%, 12.5%, 15%, 17.5%, 20%, 25%, and 50% ACN with 0.1% TEA. Eluted fractions were dried with a SpeedVac concentrator, reconstituted in 50 µL 0.1% TFA, centrifuged (20,000× *g*, 4 ${}^{\circ }$C, 30 min) and transferred to LC vials for analysis.

### 2.8. Data Acquisition and Processing

Each sample fraction was injected in triplicate for LC–MS analysis. The LC–MS system consists of a Dionex Ultimate 3000 nano LC system, a Dionex Ultimate 3000 gradient micro LC system with a WPS-3000 autosampler, and an Orbitrap Fusion Lumos mass spectrometer (Thermo Fisher Scientific, San Jose, CA, USA). A trapping nano LC setup was employed by connecting a large inner diameter (i.d.) trapping column (300 µm × 5 mm) to the nano LC column (75 µm i.d. × 65 cm, packed with 2.5 µm Waters XSelect CSH C18 material), which achieves large-capacity sample loading, on-line sample desalting and cleanup, and selective peptide delivery. Mobile phase A and B for nano LC were 0.1% FA in 2% acetonitrile (ACN) and 0.1% FA in 88% ACN. The nano LC gradient was: 4–11% B for 5 min, 11–32% B for 117 min, 32–50% for 10 min, 50–97% B for 1 min, and isocratic at 97% B for 17 min before equilibration to 4% B. Trapping column was switched on-line with the nano LC column during the first 50 min of the gradient and was switched off-line for cleanup and equilibration. MS acquisition was operated using Synchronous Precursor Selection (SPS)-MS3 method [25].

LC–MS raw files were imported to Proteome Discoverer 2.2 (Thermo Fisher Scientific, San Jose, CA, USA) for data processing. Database searching was performed by matching against Swiss-Prot Homo sapiens protein sequence database using Sequest HT. Peptides identified were validated by Percolator. Phosphosite localization was performed by ptmRS. Detailed parameters are listed as follows: Precursor Ion Tolerance, 20 ppm; Fragment Ion Tolerance, 0.02 Da; Maximum Missed Cleavages, 2; Static Modifications, cysteine (C) carbamidomethylation, peptide N-terminal TMT; Dynamic Modifications, methionine (M) oxidation, serine (S)/threonine (T) phosphorylation, N-terminal acetylation; and Percolator q-value threshold, 0.01. For quantitative phosphoproteomics, the addition of several specific parameters was necessary: ptmRS Site Probability Threshold, 50; Apply Quan Value Corrections, true; Co-Isolation Interference, 60%; Average Reporter S/N Threshold, 5; Tag Mass Tolerance, 15 ppm; Normalization Mode, none; and Scaling Mode, none.

Each phosphopeptide in the dataset is defined by a unique combination of peptide sequence and modification. For subsequent analysis, each unique phosphopeptide was considered as a separate entity, without further summarization. Each phosphopeptide was mapped to at least one protein and its corresponding gene. The normalized abundance for undetected phosphopeptides was imputed to 0. Abundance values were further processed by $log_{2}$-transformation ($log_{2}\left(x+1\right)$, where *x* is the normalized abundance value of a phosphopeptide as obtained above).

### 2.9. Differential Abundance

Differentially abundant (DA) phosphopeptides were identified using a linear model where the $log_{2}$ expression (Exp) for each phosphopeptide was expressed as a function of CyA and CXCL12 treatment, i.e., Exp∼CyA*CXCL12. We used the *lmFit* function in the R package *limma* version 3.40.6 [26], available at https://www.bioconductor.org/packages/release/bioc/html/limma.html (accessed on 27 July 2019). Samples treated with CyA are described as CyA+, and samples treated with CXCL12 are described as CXCL12+ in the text. Combinations of treatments are described as CyA+CXCL12−, CyA−CXCL12+, etc. This model was used to identify DA phosphopeptides for six contrasts: CyA+CXCL12+ vs. CyA−CXCL12+, CyA−CXCL12+ vs. CyA+CXCL12−, CyA+CXCL12+ vs. CyA+CXCL12−, CyA+CXCL12− vs. CyA−CXCL12−, CyA−CXCL12+ vs. CyA−CXCL12−, and CyA+CXCL12+ vs. CyA−CXCL12−. Contrasts for each phosphopeptide were estimated using contrasts.fit and standard errors were smoothed using the eBayes function in *limma*. DA phosphopeptides were defined as those with an absolute $log_{2}$ fold change ≥1 and a Bonferroni-adjusted *p* value <0.05. The R package *UpSetR* version 1.4.0 [27], available at https://cran.r-project.org/web/packages/UpSetR/index.html (accessed on 26 July 2019), was used to visualize the intersections between the sets of DA phosphopeptides. Samples that were cultured at 19% O${}_{2}$ and 1% O${}_{2}$ were analyzed separately because batches were separated by O${}_{2}$ levels.

### 2.10. Gene Set Enrichment Analysis and Gene Ontology Analysis

Over-representation analysis was performed on DA phosphopeptides using a one-sided hypergeometric test implemented in the R package *clusterprofiler* version 3.12.0 [28] available at https://bioconductor.org/packages/release/bioc/html/clusterProfiler.html (accessed on 3 May 2019) and gene sets from the KEGG database [29] (https://www.genome.jp/kegg/, accessed on 31 August 2021). Gene sets were considered to be significantly over-represented at a Bonferroni-adjusted *p*-value <0.05. Gene ontology (GO) enrichment analysis [30] was performed using the enrichGO function from *clusterprofiler*. Enrichment results were simplified (similarity threshold =0.5) to reduce the number of redundant GO terms in the output of this analysis.

### 2.11. Discrete-State Modeling with BONITA

A network describing the hypoxia response of B cells was assembled by combining multiple pathways from the KEGG database (Table 1, File S1). The Python tool BONITA (version 1.0) [22], available at https://github.com/Thakar-Lab/BONITA (accessed on 31 August 2021), was used with this network and protein abundance values to perform discrete-state modeling and identify logical regulatory rules for the nodes in this network. When multiple phosphopeptides were mapped to a single protein, we retained only the abundance for the phosphopeptide with the highest abundance. This abundance was assigned to the protein and used as input to BONITA. The network was perturbed in silico as described previously in [22] to identify nodes with a high influence on signal flow through this network.

## 3. Results

### 3.1. Initial Phosphoproteomic Analysis

To identify phosphorylation events regulated by cyclosporine A (CyA) and CXCL12, we performed a phosphoproteomics assay on RAMOS B cells after treatment with CyA and/or CXCL12 at 1% O2 and at 19% O${}_{2}$. As a result, a total of 17,207 phosphopeptides were quantified from 3037 proteins. Among all phosphosites characterized, 80.25% were p-serines (pS) and 7.39% were p-threonines (pT), while the remaining 12.36% are ambiguous. An average of 2.7 phosphopeptides were quantified per protein, and the TMT MS3 intensities of phosphopeptides quantified span >3 orders of magnitude. In addition, evaluation of inter- and intrabatch TMT replicates showed excellent reproducibility (data not shown). As multiple phosphopeptides were quantified for single proteins, only one phosphopeptide with the highest abundance was retained for each protein. As a result of this processing step, we considered a total of 11,652 phosphopeptides mapping to 3037 proteins for downstream analysis.

### 3.2. CyclosporineA and CXCL12 Regulate Overlapping Signal Transduction Events

**Figure 1:**
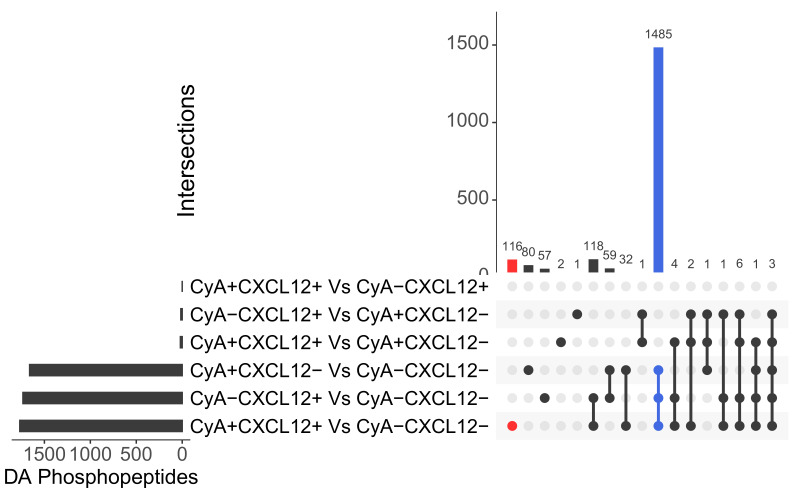
Differentially Abundant (DA) phosphopeptides are identified for multiple comparisons at 19% O${}_{2}$. UpSet plot showing the numbers of phosphopeptides that are differentially abundant in all tested contrasts and the intersections between these phosphopeptides. Empty intersections are not shown. A total of 116 phosphopeptides (labeled in red) are exclusively driven by the interaction between CyA and CXCL12. A total of 1485 phosphopeptides (labeled in blue) are driven by either CyA or CXCL12 treatment separately.

**Figure 2:**
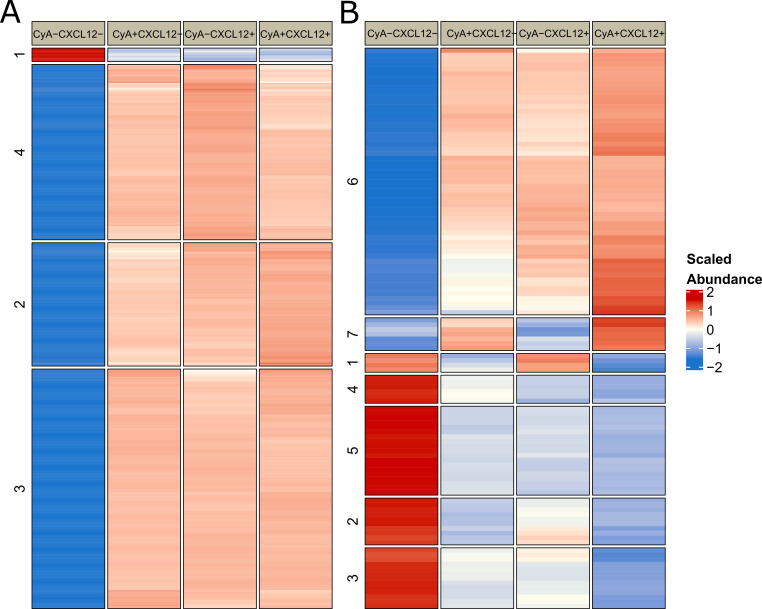
Median abundance for phosphopeptides that are differentially abundant between CyA+CXCL12+ and CyA−CXCL12− samples. (**A**) A total of 1485 differentially abundant phosphopeptides appear to be driven by either CyA or CXCL12. (**B**) A total of 116 differentially abundant phosphopeptides appear to be driven by the interaction between CyA and CXCL12.

**Figure 3:**
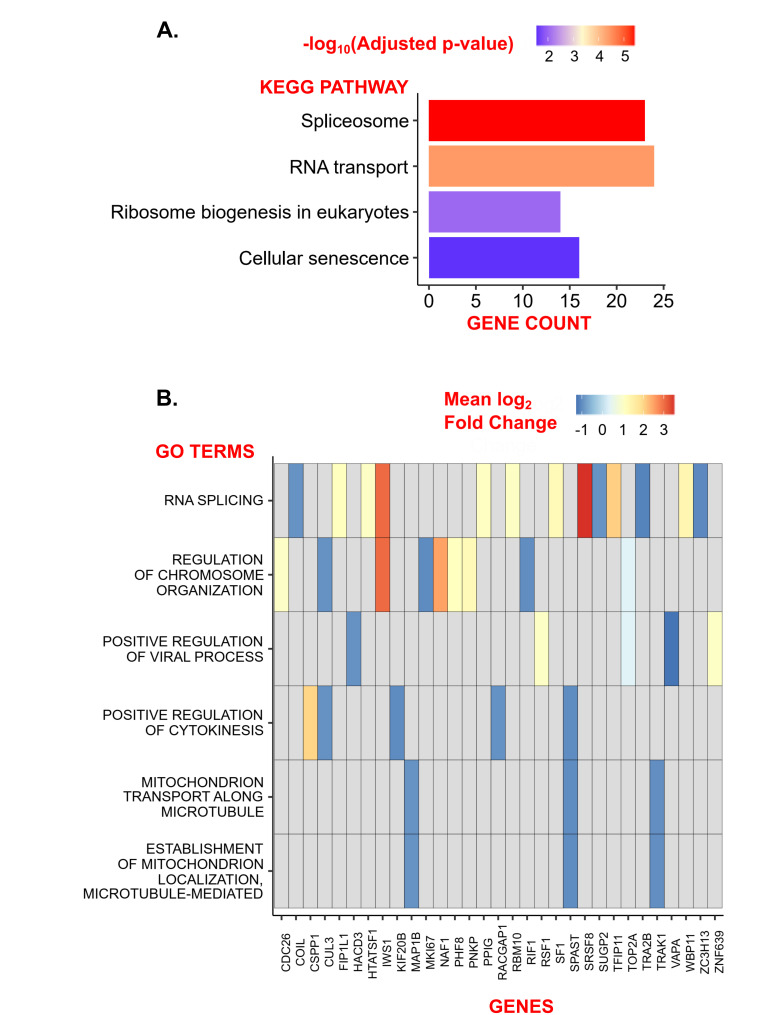
Annotations for phosphopeptides differentially abundant between CyA+CXCL12+ and CyA−CXCL12− samples. (**A**) KEGG enrichment for 1485 phosphopeptides driven by either CyA or CXCL12 treatment separately. Light gray cells [p,g] in the matrix represent the absence of a gene *g* in the KEGG pathway *p*. (**B**) GO enrichment for 116 phosphopeptides exclusively driven by the interaction between CyA and CXCL12.

Separate CyA and CXCL12 treatments independently regulated the abundance of 80 and 57 phosphopeptides, respectively (Figure 1). These differentially abundant (DA) phosphopeptides were identified by comparing CyA+CXCL12− vs. CyA−CXCL12− and CyA−CXCL12+ vs. CyA−CXCL12− samples (Methods). A total of 1485 phosphopeptides overlap between these sets of DA phosphopeptides, suggesting a similar role of CyA and CXCL12 (Figure 1 and Figure 2A). Among these phosphopeptides, 1450 were upregulated and 35 downregulated upon CyA and CXCL12 treatment (Supplementary Figure S1). Gene set over-representation analysis demonstrated that the 1485 phosphopeptide-associated genes are involved in RNA processing and in cellular senescence (Figure 3A).

The interaction between the effects of combined CyA and CXCL12 treatments at 1% O${}_{2}$ exclusively drives the abundance of 116 phosphopeptides (labeled in red in Figure 1). The remaining 1652 phosphopeptides that were DA upon CyA+ CXCL12+ treatment were also driven independently by either CyA or CXCL12 treatments (labeled in blue in Figure 1). A further examination of the abundance patterns for these 116 phosphopeptides suggests that independent CyA or CXCL12 treatments change abundance in the same direction; however, joint treatment has a larger impact on the change in abundance (e.g., clusters 3 and 4 in Figure 2B). Gene ontology analysis revealed that the 116 phosphopeptide levels exclusively driven by the interaction between CyA and CXCL12 treatment are involved mainly in microtubule-mediated processes, providing a link between the combined CyA and CXCL12 treatment and cellular motility (Figure 3B). This phosphoprotein set was largely downregulated in CyA+CXCL12+ samples, suggesting a mechanism for the observed decrease in cellular motility. A similar comparison between CyA+CXCL12+ and CyA−CXCL12− samples grown at 19% O${}_{2}$ revealed only 30 DA phosphopeptides (Supplementary Figure S2). None of these were exclusively driven by the interaction between CyA and CXCL12 treatments. This suggests that the synergistic interaction between CyA and CXCL12 and the subsequent effects on cellular motility are exclusively observed in B cells under hypoxic conditions.

### 3.3. Kinase–Substrate Enrichment Analysis

**Figure 4:**
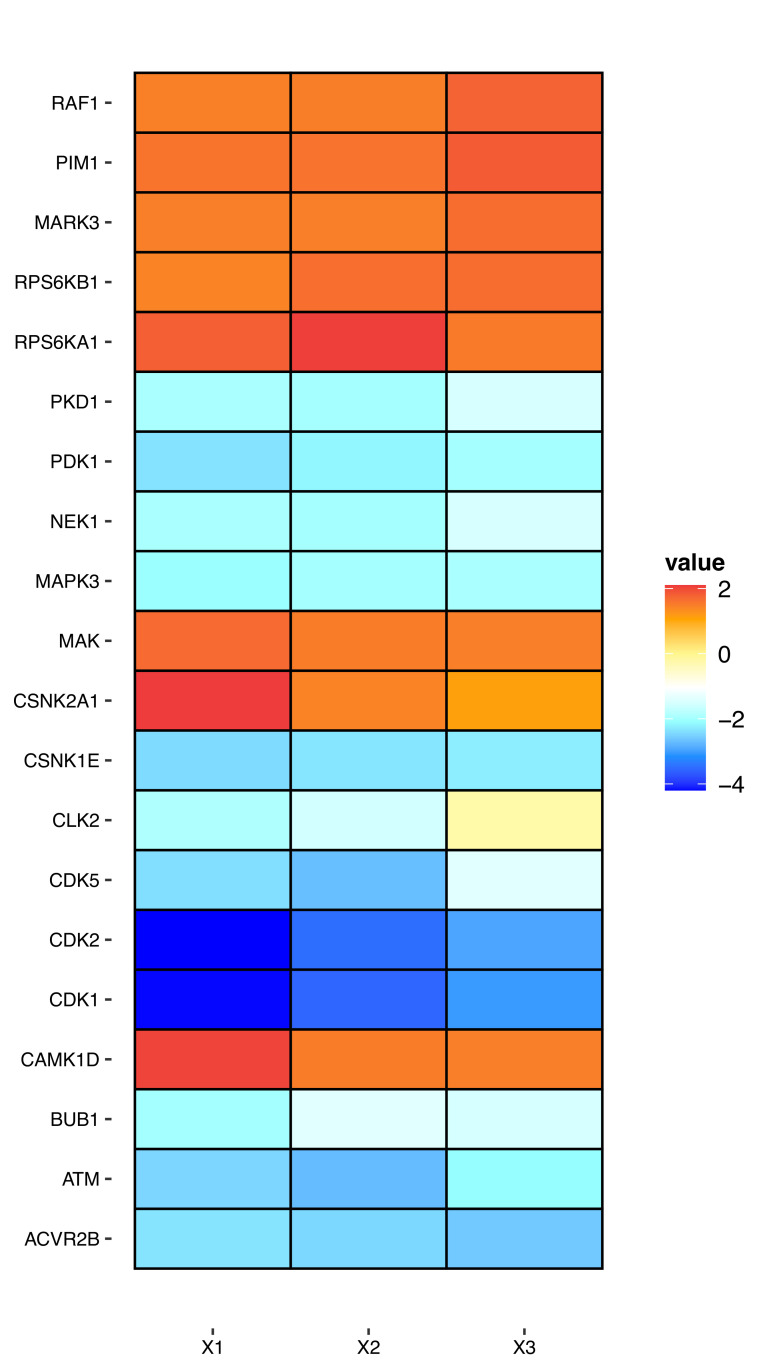
Kinase substrate analysis. The three columns of the heat map refer to the following three sets of comparisons: X1. CyA+CXCL12+ vs. CyA−CXCL12−; X2. CyA−CXCL12+ vs. CyA−CXCL12−; and X3. CyA+CXCL12− vs. CyA−CXCL12−.

In order to further investigate which upstream kinases might be participating in the global phosphoproteomic changes after CyA and/or CXCL12 treatment, the 1485 DA phosphopeptides regulated by both CyA and CXCL12 and their corresponding fold changes and p-values from three comparisons depicted in Figure 1 were imported into the KSEA App (https://casecpb.shinyapps.io/ksea/ accessed on 31 August 2021) for a kinase–substrate enrichment analysis. The analysis revealed upregulated activities of RAF1, PIM1, MARK3, MAK, CSNK2A1, CAMK1D, and two RPS6Ks (A1 and B1) and downregulated activities of PKD1, PDK1, NEK1, MAPK3, CSNK1E, CLK2, BUB1, ATM, ACVR2B, as well as three CDKs (CDK1, CDK2, and CDK5) (Figure 4. Moreover, KSEA of the 116 jointly regulated by CyA and CXCL12 phosphopeptides suggested upregulated activities of CSNK2A1 as well as downregulated activities of CLK2, MAPK14, and two isoforms of novel PKCs (PRKCE and PRKCD).

### 3.4. Discrete-State Modeling with BONITA Links the HIF-1α-Mediated Hypoxia Response to Remodeling of the Actin Cytoskeleton

**Figure 5:**
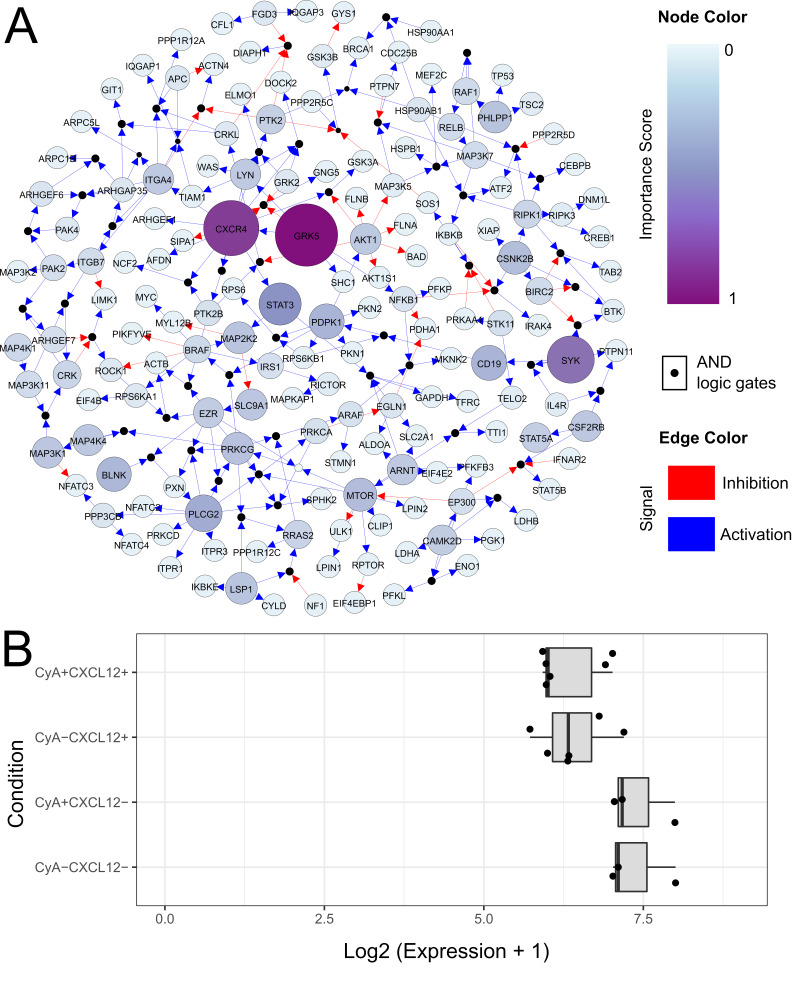
Differential abundance and discrete-state modeling show a potential role for LSP1 in the interaction between CyA and CXCL12. (**A**) A logic rule model of HIF1A-mediated hypoxia response and cytoskeletal rearrangement. A random subset of logic rules inferred by BONITA is shown. Nodes (except black nodes) represent proteins and black nodes represent inferred ’and’ logic gates. Incoming edges represent ’OR’ logic gates. The size of nodes is proportional to their importance score as calculated by BONITA, i.e., it is proportional to their influence over signal flow through the network. Nodes representing proteins are colored according to the fold change between CyA+CXCL12+ and CyA−CXCL12− samples as shown in the legend. Edges are colored by activation or inhibition signal as shown in the legend. (**B**) Abundance of an LSP1 phosphopeptide that is exclusively differentially abundant in the CyA+CXCL12+ vs. CyA−CXCL12− comparison at 1% O${}_{2}$.

To investigate the regulation between the HIF1α-mediated hypoxia response and changes in cellular motility, we assembled a directed protein–protein interaction network using KEGG pathways linked to HIF1α-mediated downstream signaling processes (Table 1, Supplementary File S1). This network was significantly dysregulated between CyA+CXCL12+ and CyA-CXCL12- samples grown at 1% O${}_{2}$ (*p* = 2.81677 × 10${}^{-5}$) when analyzed by BONITA. Figure 5A shows the topology of this network, the inferred logic rules underlying regulation, and the calculated importance scores. The BONITA importance scores rank the proteins with pivotal roles in signal flow through the network. The most influential nodes in the network were GRK5, CXCR4, SYK, and STAT3 (Table 2, Supplementary File S2). GRK5 phosphorylates CXCR4 and promotes its internalization in response to activation by CXCL12 [31]. GRK5 and CXCR4 were both upregulated in CyA+CXCL12+ samples grown at low O${}_{2}$ and are both highly connected, indicating that they modulate an array of downstream signaling events. LSP1 had a high importance score and mediates actin cytoskeletal remodeling by inhibiting the action of PIKFYVE via RRAS2. The inhibition of PIKFYVE has been shown to block chemotaxis in neutrophils [32]. RRAS2 also modulates the inflammation response by inhibiting NFKB1 and MAP3K7. We found that LSP1 also modulates downstream programs of post-translational modification/phosphorylation by activating ARAF, MAP2K2, and PPP1R12C. The high relative abundance of LSP1 in the CyA+CXCL12+ vs. CyA−CXCL12− comparison (Figure 5B) and the corresponding changes in the abundances of its downstream nodes prompted a further investigation of the role of LSP1 in modulating changes in the cytoskeleton.

### 3.5. LSP1 May Mediate Interaction between CyA and CXCL12

As our previous work has suggested that cytoskeletal components play a critical role in CyA and O${}_{2}$ modulated B cell migration, we chose to focus further analysis specifically on LSP1. The cluster 1 of genes in Figure 1 has a higher abundance in CyA−CXCL12− and a significantly decreased abundance in CyA+CXCL12+ samples. This cluster includes the gene LSP1, which is involved in the arrangement of the cytoskeleton via f-actin binding, causing actin filiment polymerization and affecting cellular migration. We examined the abundance of all phosphopeptides corresponding to the LSP1 gene (Supplementary Figure S3A). Figure 5B shows the abundance of a phosphopeptide corresponding to LSP1 that is exclusively differentially expressed in the CyA+CXCL12+ vs. CyA−CXCL12− contrast at 1% O${}_{2}$. This phosphopeptide has higher abundance in the absence of CXCL12 (i.e., in the samples denoted as CyA+CXCL12− and CyA−CXCL12−) and was also differentially abundant between CyA−CXCL12− samples at 19% O${}_{2}$ and 1% O${}_{2}$ (*t*-test, p<0.05, Supplementary Figure S3B).

### 3.6. Human B Cell LSP1 Protein Amounts Are Increased at Low O${}_{2}$ Levels and Attenuated by CyA Treatment

**Figure 6:**
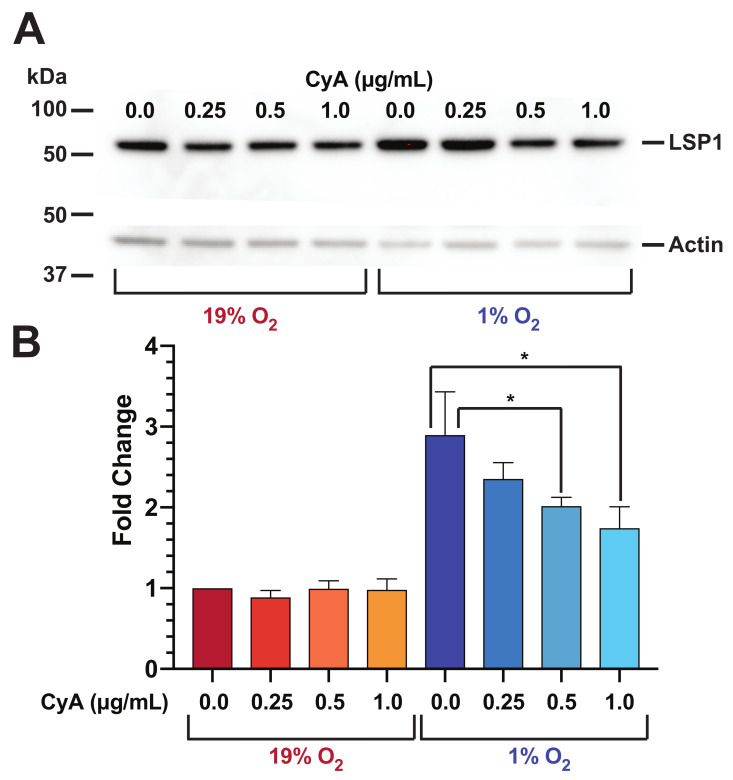
Human B cell LSP1 protein expression is modulated by both O${}_{2}$ levels and CyA treatment. (**A**) Human RAMOS cells were incubated at the indicated O${}_{2}$ levels for 24 h in the absence or presence of graded CyA concentrations. Whole-cell lysates were then subject to Western analysis for LSP1 and actin. Shown is a representative image (*n* = 3). (**B**) LSP1 protein levels were normalized to actin amounts and fold change relative to 19% O${}_{2}$ without CyA calculated. Shown are the mean fold changes ± s.d. for 3 independent experiments. (*t*-test, * *p* value <0.05). Western images presented in Figure S4.

We first wanted to assess the effect different O${}_{2}$ levels have on total LSP1 protein amounts. We incubated RAMOS cells at 19% or 1% O${}_{2}$ levels for 24 h, and whole-cell lysates were analyzed by LSP1 Western blotting. Figure 6 shows the incubation of RAMOS B cells at 1% O${}_{2}$ conditions results in a 2.8 fold increase in total LSP1 protein as compared with 19% conditions (*t*-test, p<0.05). In addition, we assessed the effect CyA treatment has on LSP1 protein levels at both 19% and 1% O${}_{2}$ levels. As is evident from Figure 6 at 19% O${}_{2}$ levels, we did not observe a significant affect of CyA on LSP1 protein levels. However, treatment of RAMOS cells with increasing amounts of CyA at 1% O${}_{2}$ results in a progressive decrease in LSP1 protein levels. Specifically, we observed a 1.4 and 1.6 fold decrease for 0.5 and 1.0 µg/mL concentrations of CyA, respectively (*t*-test, p<0.05).

### 3.7. LSP1 Protein Levels Directly Affect O${}_{2}$-Dependent Chemokine Receptor Hypo-Responsiveness

**Figure 7:**
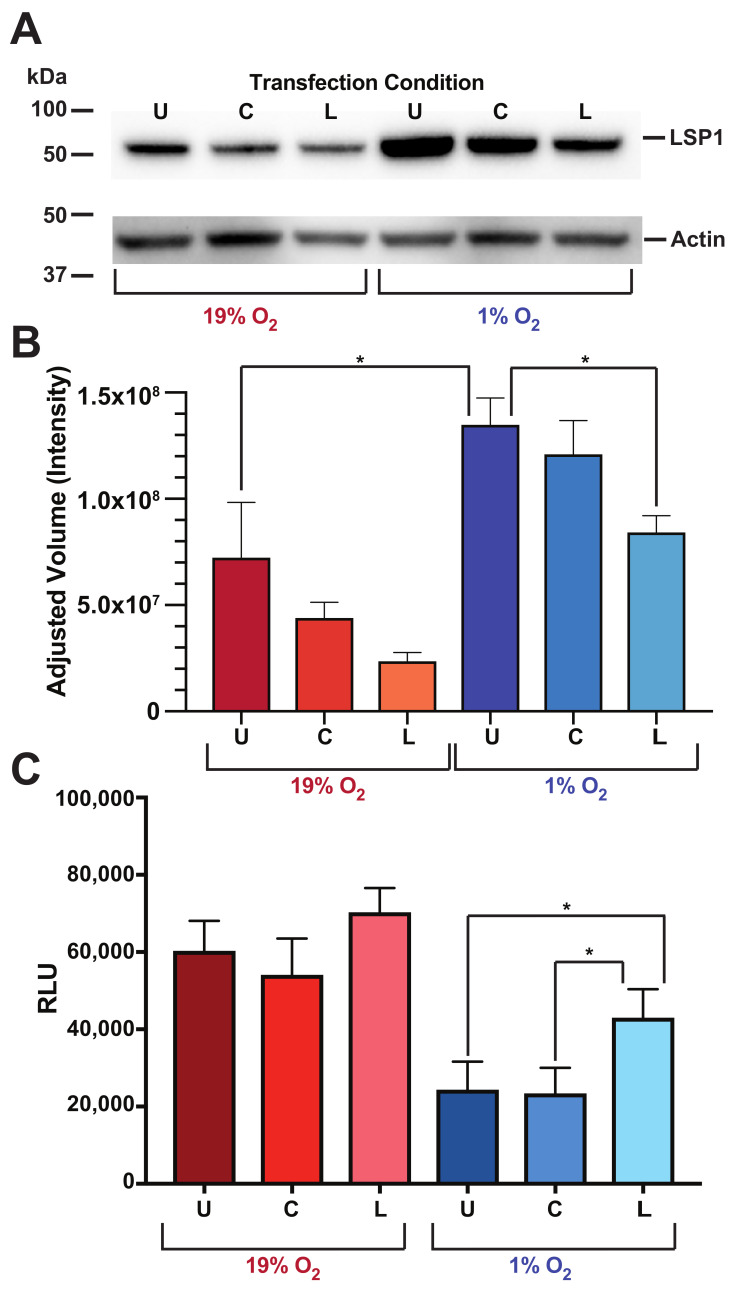
Human B cell LSP1 protein expression directly modulates chemotactic capacity. Human RAMOS cells were transfected with an shRNA corresponding to human LSP1 (L), a control shRNA (C) or left untransfected (U). Cells were incubated at the at the indicated O${}_{2}$ levels for 24 h. (**A**) Whole-cell lysates were then subject to Western analysis for LSP1 and actin. Shown is a representative image (*n* = 3). (**B**) LSP1 protein levels were normalized to actin amounts and fold change relative to untransfected 19% O${}_{2}$ calculated. Shown are the mean fold changes ± s.d. for 3 independent experiments. (*t*-test, * *p* value <0.05). (**C**) Alternatively, cells were subjected to the chemotaxis assay (two-way ANOVA, * *p* value <0.008). Western images presented in Supplementary Figure S4.

To directly assess the role of LSP1 in O${}_{2}$-dependent chemokine receptor responsiveness, we generated a RAMOS cell LSP1 knockdown (KD) using an LSP1 shRNA lentiviral vector. As is evident from Figure 7, our transcfection of RAMOS cell with the LSP1 shRNA vector results in a significant decrease in LSP1 protein levels at the 1% O${}_{2}$ conditions (*t*-test, p<0.05) but not at 19% O${}_{2}$. We next assessed the ability of LSP1 KD RAMOS cells to migrate in response to a chemokine gradient. As we have previously shown, untransfected RAMOS cells migrate in responses to a CXCL12 gradient at 19% O${}_{2}$ levels, with migration significantly decreased at low, 1% O${}_{2}$. Interestingly, genetic inhibition of LSP1 expression by the shRNA increases the chemotactic capacity at both 19% and 1% O${}_{2}$ levels. However, only the increase at the 1% O${}_{2}$ level is statistically significant (two-way ANOVA, p<0.008). Importantly, no significant difference in chemotaxis at either O${}_{2}$ level was seen in RAMOS cells transfected with a control shRNA with a sequence that does not target any know mammalian gene. These results indicate that LSP1 protein levels are sufficient to alter chemotactic capacity in the RAMOS human B cells.

## 4. Discussion

Hypoxic signaling plays a significant role in the development of human B cells’ responses to infection and vaccination [33,34,35,36,37]. B cells encounter a variety of O${}_{2}$ levels as they migrate to, from, and within secondary lymphoid organs [1,2]. Of particular interest, activated B cells encounter low O${}_{2}$ concentrations as they differentiate within the hypoxic germinal center (GC) environment, which has been identified as an important molecular switch controlling many aspects of B cell activation, differentiation, and migration [1,3,4,6,21,38]. Indeed, germinal center (GC) formation itself has been tied to hypoxia; B cell follicles proximal to high O${}_{2}$ blood vessels are inhibited from forming GCs [6]. In addition, somatic hypermutation, which occurs within the GC, has also been show to be influenced by hypoxia [4]. We have previously shown a negative correlation between low O${}_{2}$ tension and B cell migratory capacity and that the calcineurin inhibitor, cyclosporine A (CyA), disrupts the B cells’ ability to functionally react to altered O${}_{2}$ conditions [3]. This O${}_{2}$-dependent molecular switch is centrally controlled by the transcription factor HIF-1α. Indeed, our results combined with those of others, strongly implicate migration as at least one mechanism by which O${}_{2}$ levels can affect immunological function. However, the molecular components responsible for altering B cell chemotactic capacity in response to O${}_{2}$ changes have not previously been well-defined. This study has done so using a phosphoproteomics approach to identify phosphorylated proteins that functionally contribute to hypoxia-induced modulation of CXCE4-dependend B cell chemotaxis.

Accurate modulation of B cell chemotaxis is consequential as B cell migration between different lymphoid organs, and internal anatomic areas within them, is required for differentiation and maturation [39,40]. As such, the ability for B cells to correctly modulate their migration is a crucial factor in an effective immune response [41]. Indeed, GC B cell location itself is highly dependent on CXCR4 and SIP1 modulation [42]. Importantly, perturbation of CXCR4 signaling disrupts coordinated spatial localization of B cells between the GC light and dark zones, resulting in decreased somatic hypermutation and Ig affinity maturation [7]. Our phosphoproteomic analysis identified 1989 phosphopeptides that are differentially phosphorylated in human B cells under low O${}_{2}$ conditions. Of these, 80 phosphopeptides are exclusively modulated by the addition of CyA and 116 phosphopeptides are exclusively regulated by the combined interaction of CyA and CXCL12. Importantly, this analysis identified LSP1 as specifically regulated by the combination of CyA and CXCL12, an observation confirmed by shRNA knockdown experiments.

Our findings build upon previous evidence showing that LSP1 modulates migration in lymphocytes among other immune cells. Using transwell migration assays, Hwang et al. found that LSP1 attenuates chemokine-driven migration in murine CD4+ T cells, and Jongstra-Bilen et al. found that LSP1 possesses the same function in neutrophils [43,44]. Mechanistically, LSP1 has been observed as having two functions: it acts both as an F-actin bundling protein and as a scaffold for the targeted localization of kinases to the cytoskeleton. More specifically, LSP1 binds PKCβI directly, localizing a complex of KSR, MEK1, and ERK2 to the cytoskeleton of W10 B cells [45]. For LSP1 to recruit PKCβI, LSP1 must first be O-GlcNAcylated by O-GlcNAc transferase, which is upregulated by anti-IgM F(ab${}^{\prime }$)2-induced BCR activation [46]. Linking this finding to B cell chemotaxis, IgM stimulation of chronic lymphocytic leukaemia (CLL) B cells decreased CXCL12 chemotaxis [47]. Given the previous evidence for LSP1’s role in the phosphorylation events and localization pertinent to chemotaxis, our data strongly support the importance of LSP1 in attenuating chemotaxis. We show that LSP1 expression increases with low oxygen tension, that low oxygen attenuates RAMOS B cell chemotaxis, and that knocked-down translational expression of LSP1 significantly increases chemotaxis. These findings strongly support a central role for LSP1 in attenuating CXCR4−CXCL12−driven chemotaxis.

Calcineurine inhibitors (CNIs), such as CyA, are often utilized to prevent allograft rejection in solid-organ transplant recipients [8]. However, systemic immunosuppression frequently results in hypogammaglobulinemia, leading to reduced protective IgG responses after vaccinations such as influenza [10,11]. Importantly, it has become increasingly evident that immune responses to SARS-CoV-2 vaccines are similarly impaired in transplant patients taking calcineurin inhibitors ( [48,49,50,51], resulting in increased morbidity and mortality associated with SARS-CoV-2 infection [52,53]. While this phenomenon is largely thought to be due to CyA effects blocking CD4 T cell activation, indirectly altering B cell responses [12], our past [3] and current work suggest that CyA also inhibits B cell immune responses by LSP1 modulation of O${}_{2}$-dependent B cell migratory capacity. This is the first time, to our knowledge, that a CyA-modulated cytoskeletal component, LSP1, was shown to directly effect B cell migration by disrupting the natural O${}_{2}$-sensing molecular switch, destabilizing HIF-1α, and allowing B cells to preserve CXCR4 responsiveness at low (<1%) O${}_{2}$ levels. Future work will explore if this is a target-able pathway component, by which CyA’s immunosuppressive function may be transiently altered to enhance vaccine responses in immunosuppressed individuals.

## Figures and Tables

**Table 1:** KEGG Pathways ${}^{†}$ Used to Construct a Network Linking HIF1α and Cytoskeletal Changes.

KEGG Pathway	KEGG Code
MAPK signaling	hsa04010
Chemokine signaling	hsa04062
NF-kappa B signaling	hsa04064
HIF-1 signaling	hsa04066
mTOR signaling	hsa04150
PI3K-Akt signaling	hsa04151
VEGF signaling	hsa04370
Cell adhesion	hsa04514
C-type lectin receptor signaling	hsa04625
JAK-STAT signaling	hsa04630
TNF signaling	hsa04668
Leukocyte transendothelial migration	hsa04670
Regulation of actin cytoskeleton	hsa04810

† All pathways are Homo sapiens (human).

**Table 2:** Top 15 High-Importance Nodes from the HIF1α/Cytoskeletal Changes Network, as identified by BONITA ${}^{†}$.

Node	Importance Score from BONITA
GRK5	1.00
CXCR4	0.83
SYK	0.63
STAT3	0.51
PLCG2	0.38
CD19	0.34
PDPK1	0.34
BLNK	0.33
MTOR	0.30
CSNK2B	0.29
PRKCG	0.27
PHLPP1	0.27
MAP2K2	0.27
LSP1	0.26

† The complete list of nodes and importance scores is available in Supplementary File S2.

## Data Availability

The mass spectrometry phosphoproteomics data generated in this study have been deposited to the ProteomeXchange Consortium via the PRIDE [54] partner repository with the dataset identifier PXD036167.

## Supplementary Materials

The following supporting information can be downloaded at: https://www.mdpi.com/2075-1729/12/8/1284/s1. Supplementary Figure S1: Upregulated (A) and downregulated (B) Differentially Abundant (DA) phosphopeptides across all tested comparisons at 1% O${}_{2}$. Phosphopeptides labeled in red are exclusively driven by the interaction between CyA and CXCL12. Phosphopeptides labeled in blue are driven by either CyA or CXCL12 treatment separately. Empty intersections are not shown; Supplementary Figure S2: Differentially Abundant (DA) phosphopeptides identified in the CyA+CXCL12+ vs. CyA−CXCL12− contrast at 19% O${}_{2}$. (A) UpSet plot showing the numbers of phosphopeptides that are differentially expressed in all tested contrasts and the intersections between these phosphopeptides. Empty intersections are not shown. A total of 30 phosphopeptides are DA between CyA+CXCL12+ and CyA−CXCL12− contrast at 19% O${}_{2}$. (B) Median abundance for the 30 phosphopeptides that are differentially abundant between CyA+CXCL12+ and CyA−CXCL12− samples at 19% O${}_{2}$; Supplementary Figure S3: Abundance of phosphopeptides corresponding to LSP1 across all samples. (A) Abundance of all phosphopeptides corresponding to LSP1. The row labeled with a single asterisk (*) corresponds to a phosphopeptide that is differentially abundant between CyA+CXCL12+ and CyA−CXCL12− but is not exclusively differentially abundant in this contrast. The row labeled with double asterisks (’**’) corresponds to a phosphopeptide that is exclusively differentially abundant in the CyA+CXCL12+ vs. CyA−CXCL12− comparison. (B) Abundance of a phosphopeptide corresponding to LSP1 across all samples grown at 1% O${}_{2}$, and CyA−CXCL12− samples grown at 19% O${}_{2}$. This phosphopeptide is marked with a double asterisk (**) in panel (A); Supplementary Figure S4: Western blot images. (A) Blots from Figure 6. (B) Blots from Figure 7; Supplementary File S1: Network describing the hypoxia response of B cells assembled by combining KEGG pathways listed in Table 2; Supplementary File S2: Network nodes from File S1 along with their importance scores as calculated by BONITA.
