# Supplementary material for: Cyclosporine A Modulates LSP1 Protein Levels in Human B Cells to Attenuate B Cell Migration at Low O2 Levels
Source: Life (Basel). 2022 Aug 22;12(8):1284. doi: 10.3390/life12081284 (PMC9410508; doi:10.3390/life12081284)
Supplement: Supplementary file 1 [file life-12-01284-s001.zip › life-1839738-Supplementary figures.pdf]

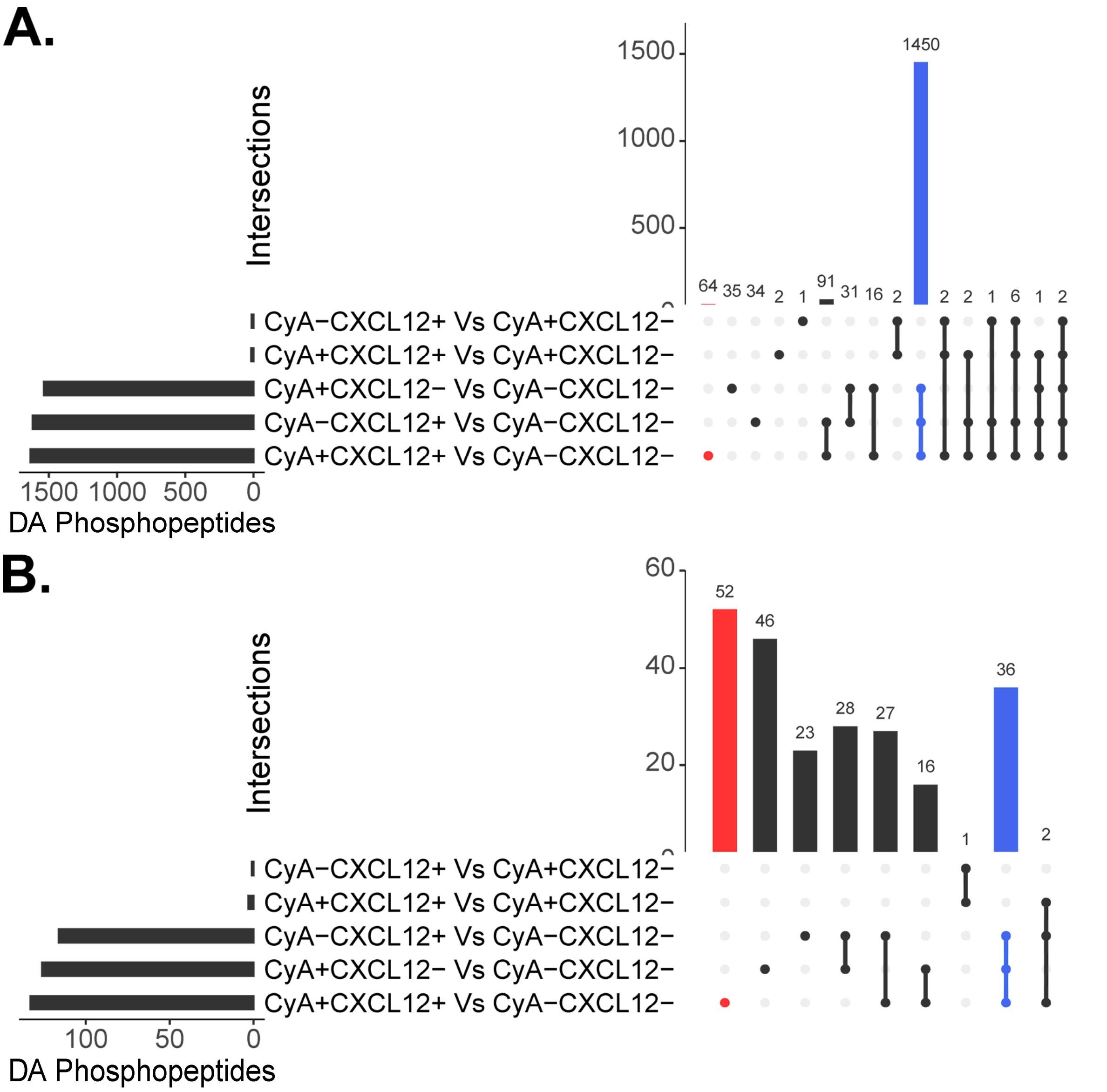

Supplementary Figure S1. Upregulated (**A**) and downregulated (**B**) Differentially Abundant (DA) phosphopeptides across all tested comparisons at 1% O_2. Phosphopeptides labeled in red are exclusively driven by the interaction between CyA and CXCL12. Phosphopeptides labeled in blue are driven by either CyA or CXCL12 treatment separately. Empty intersections are not shown.

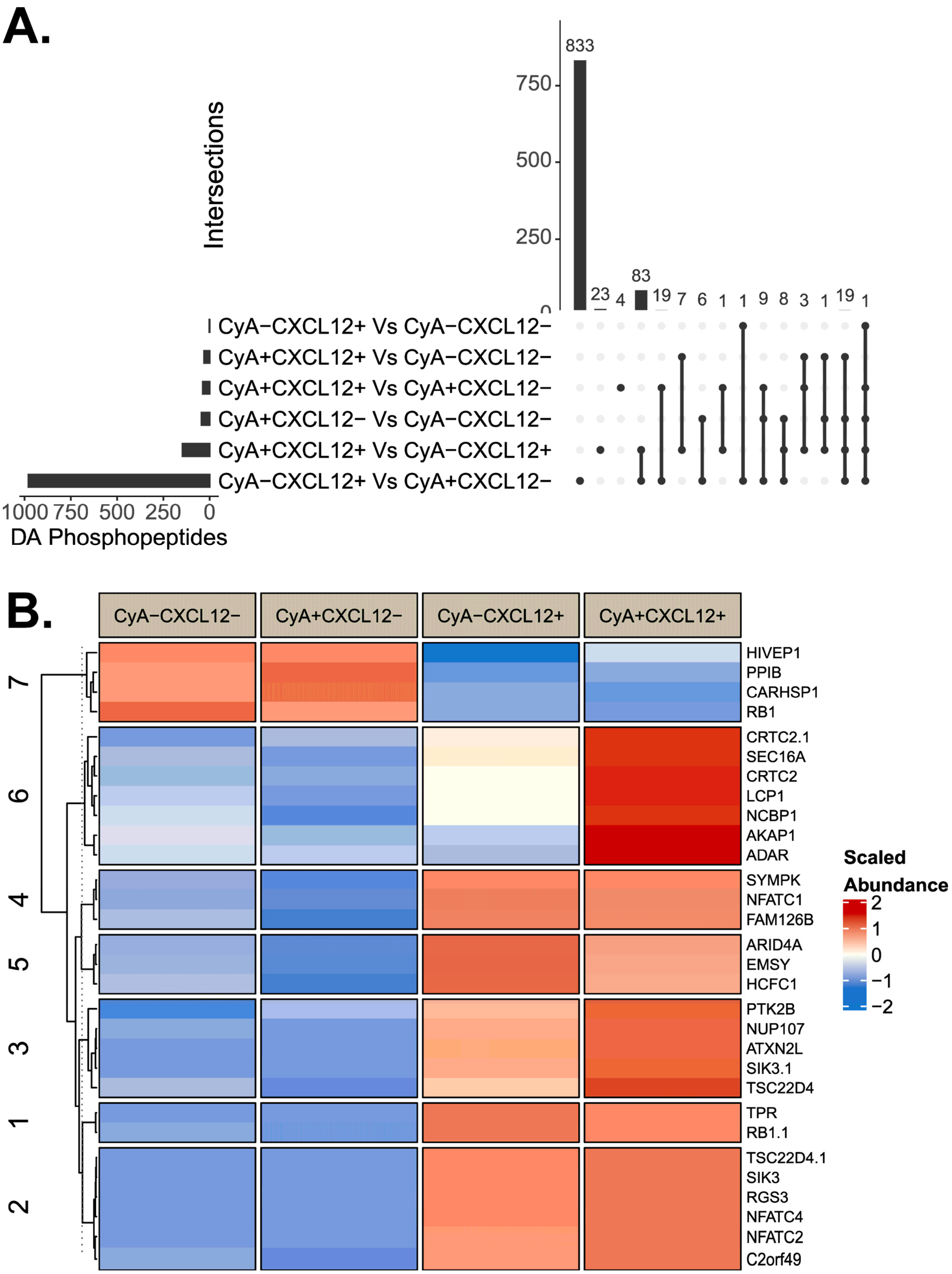

Supplementary Figure S2. Differentially Abundant (DA) Phosphopeptides identified in the CyA+CXCL12+ vs. CyA−CXCL12− contrast at 19% O_2. (**A**) UpSet plot showing the numbers of phosphopeptides that are differentially expressed in all tested contrasts, and the intersections between these phosphopeptides. Empty intersections are not shown. 30 phosphopeptides are DA between CyA+CXCL12+ and CyA−CXCL12− contrast at 19% O_2. (**B**) Median abundance for the 30

phosphopeptides that are differentially abundant between CyA+CXCL12+ and CyA−CXCL12− samples at 19% O_2.

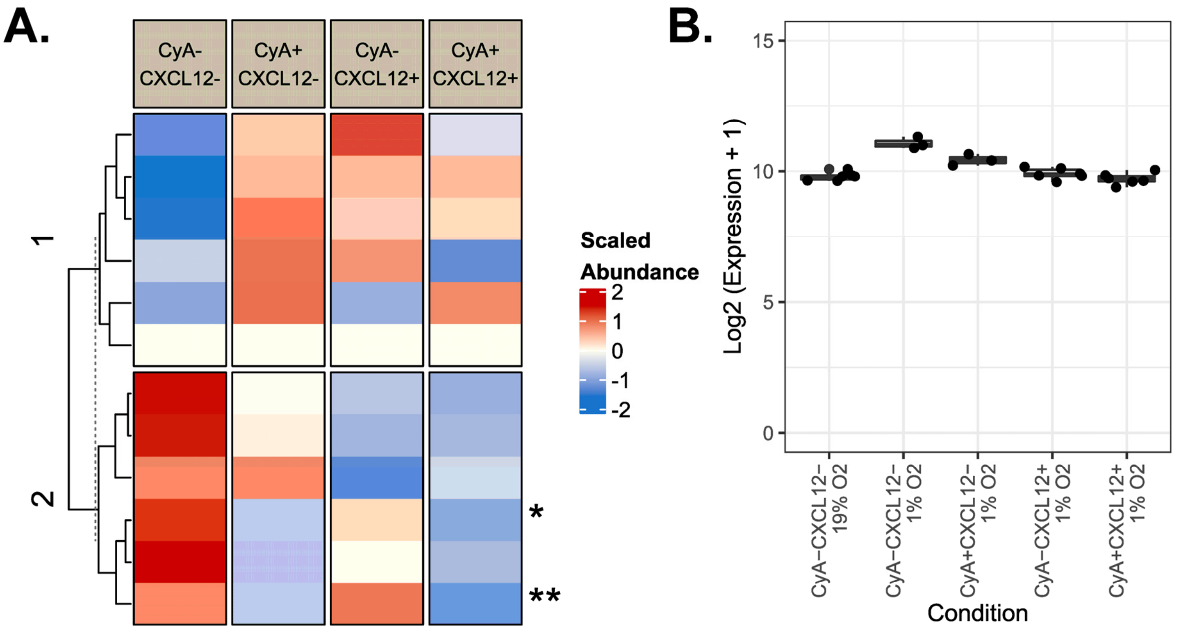

Supplementary Figure S3. Abundance of Phosphopeptides Corresponding to LSP1 Across all Samples. (**A**) Abundance of all phosphopeptides corresponding to LSP1. The row labeled with a single asterisk (*) corresponds to a phosphopeptide that is differentially abundant between CyA+CXCL12+ and CyA−CXCL12− but is not exclusively differentially abundant in this contrast. The row labeled with double asterisks ("**") corresponds to a phosphopeptide that is exclusively differentially abundant in the CyA+CXCL12+ vs. CyA−CXCL12− comparison. (**B**) Abundance of a phosphopeptide corresponding to LSP1 across all samples grown at 1% O_2, and CyA−CXCL12− samples grown at 19% O_2. This phosphopeptide is marked with a double asterisk (**) in panel (**A**).

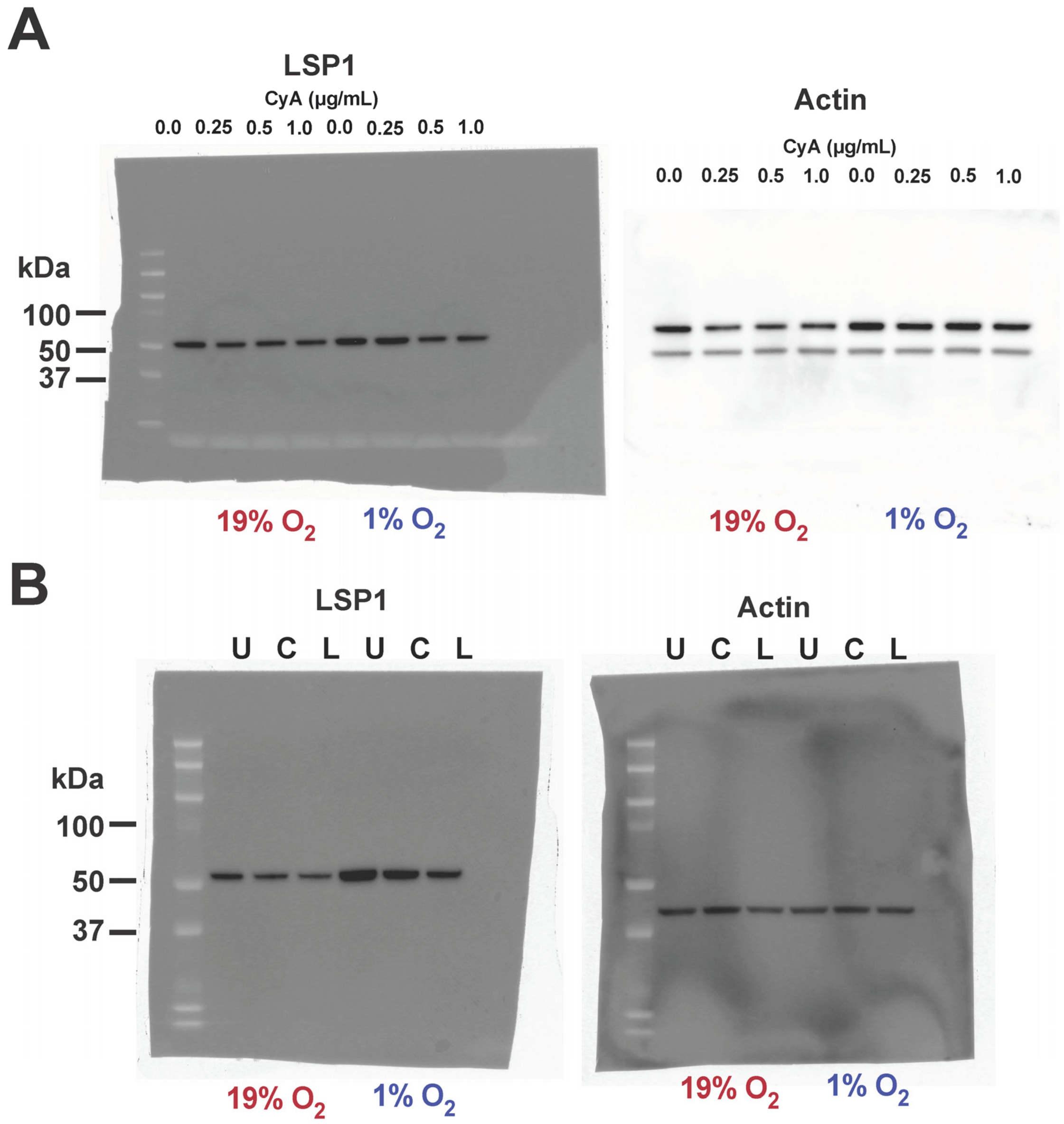

Supplementary Figure S4. Western blot images. (**A**) Blots from Figure 6. (**B**) Blots from Figure 7.
